# In silico characterization of cell–cell interactions using a cellular automata model of cell culture

**DOI:** 10.1186/s13104-017-2613-x

**Published:** 2017-07-14

**Authors:** Takanori Kihara, Kosuke Kashitani, Jun Miyake

**Affiliations:** 1 Department of Life and Environment Engineering, Faculty of Environmental Engineering, The University of Kitakyushu, 1-1 Hibikino, Wakamatsu, Kitakyushu, Fukuoka 808-0135 Japan; 2 Department of Mechanical Science and Bioengineering, Graduate School of Engineering Science, Osaka University, 1-3 Machikaneyama, Toyonaka, Osaka 560-8531 Japan

**Keywords:** Cell proliferation, Cell–cell adhesion, Cell–cell contact inhibition, Cellular automata, Cell assay system

## Abstract

**Background:**

Cell proliferation is a key characteristic of eukaryotic cells. During cell proliferation, cells interact with each other. In this study, we developed a cellular automata model to estimate cell–cell interactions using experimentally obtained images of cultured cells.

**Results:**

We used four types of cells; HeLa cells, human osteosarcoma (HOS) cells, rat mesenchymal stem cells (MSCs), and rat smooth muscle A7r5 cells. These cells were cultured and stained daily. The obtained cell images were binarized and clipped into squares containing about 10^4^ cells. These cells showed characteristic cell proliferation patterns. The growth curves of these cells were generated from the cell proliferation images and we determined the doubling time of these cells from the growth curves. We developed a simple cellular automata system with an easily accessible graphical user interface. This system has five variable parameters, namely, initial cell number, doubling time, motility, cell–cell adhesion, and cell–cell contact inhibition (of proliferation). Within these parameters, we obtained initial cell numbers and doubling times experimentally. We set the motility at a constant value because the effect of the parameter for our simulation was restricted. Therefore, we simulated cell proliferation behavior with cell–cell adhesion and cell–cell contact inhibition as variables. By comparing growth curves and proliferation cell images, we succeeded in determining the cell–cell interaction properties of each cell. Simulated HeLa and HOS cells exhibited low cell–cell adhesion and weak cell–cell contact inhibition. Simulated MSCs exhibited high cell–cell adhesion and positive cell–cell contact inhibition. Simulated A7r5 cells exhibited low cell–cell adhesion and strong cell–cell contact inhibition. These simulated results correlated with the experimental growth curves and proliferation images.

**Conclusions:**

Our simulation approach is an easy method for evaluating the cell–cell interaction properties of cells.

## Background

Cell culture techniques enable us to directly observe cells in vitro. Today, we can culture many types of cells including triploblastic, stem, and cancer cells. These cells can be viable and can proliferate under 2 dimensional (2D) cell culture conditions. Cells demonstrate their unique characteristics under such conditions; in particular, proliferation is one of the unique characteristics of cultured cells. During cell proliferation, cells not only divide but also move and interact with each other. These elementary steps of cell proliferation demonstrate the characteristics of different cell types [1].

Cell culture is often used in cell assay systems, such as in evaluating the effect of drugs on cancer cells [2, 3]. Considering the necessity for reduction of animal experiments, the demand for in vitro cell assay systems for drug and cosmetic development has increased [4, 5]. The parameters available for evaluation from the cell assay system are cell growth rate, motility, and gene expression [6–9]. On the other hand, cell–cell interactions are also important parameters for cell characterization. Epithelial and endothelial cells show high intercellular adhesion and they ultimately form tight junctions [10, 11]. Fibroblasts and smooth muscle cells adhere to each other and form cell aggregates [12, 13]. In the process of epithelial-to-mesenchymal transformation, which is essential for animal and cancer development, intercellular adhesiveness decreases [14]. Increasing the density of endothelial or smooth muscle cells increased cell–cell contact and decreased cell spreading, leading to growth arrest [15]. Furthermore, the homotypic intercellular adhesive forces between ectoderm, mesoderm, and endoderm cells are different in each other [16]. Although these cell–cell interactions are important properties of cells, they are hard to evaluate in standard cell assay systems. Therefore, simple methods for evaluating cell–cell interaction properties are required for the development of cell assay systems for drug screening.

Mathematical modeling and computational simulation using differential equations and cellular automata assume an important role in experimental modeling to study a variety of biological events [17]. Modeling is performed by abstracting some characteristic factors from the biological events, and the simulation displays the reconstruction results when those factors are used. Thus, modeling and computational simulation can help us to understand how certain biological processes occur, as well as inform us of the critical factors involved in these biological processes. There are many cellular automata models that can describe the population dynamics of cultured cells [1, 18–22]. These models capture the features of the proliferation process and include important parameters (cell size, seeding density, spatial distribution of cells, migration, and oxygen density) necessary for prediction of cell population behavior. Furthermore, there are some cellular automata models that include cell–cell adhesion effects to simulate cultured cells in 2D and 3D [19, 20, 23]. Therefore, using these models, we can estimate the cell–cell interaction properties of cultured cells.

In this study, we developed a system for estimating the cell–cell interactions of cultured cells using a cellular automata model. To estimate the interaction parameters, we focused on images of cells in 2D cell culture because cell–cell interactions, especially cell–cell adhesion and cell–cell contact inhibition (of proliferation), affect the formation of cell aggregates. We obtained cultured cell images over time, and then assessed these images for corroboration with the cellular automata system. Using the cell growth rate and seeding density, which were determined from the images, we succeeded in estimating the cell–cell adhesion and cell–cell contact inhibition parameters. This method is useful for estimating these cell–cell interaction properties. This method could therefore be used as a new cell assay system for analysis of cell–cell interactions during drug screening.

## Methods

### Materials

Male Fisher 344 rats were purchased from Japan SLC (Shizuoka, Japan). The rat aorta smooth muscle cell line, A7r5, was obtained from DS Pharma Biomedical Co. Ltd (Osaka, Japan). The human cervical cancer cell line, HeLa, and the human osteosarcoma cell line, HOS, were obtained from the Health Science Research Resources Bank (Osaka, Japan). Cell culture medium was purchased from Sigma-Aldrich (St. Louis, MO). Fetal bovine serum (FBS) was purchased from JRH Biosciences (Lenexa, KS). Antibiotics were purchased from Life Technologies Japan Ltd. (Tokyo, Japan). Other reagents were purchased from Wako Pure Chemical Industries Ltd. (Osaka, Japan), Sigma-Aldrich, and Life Technologies Japan Ltd.

### Preparation and culture of rat mesenchymal stem cells

Rat mesenchymal stem cells (MSCs) were isolated and primarily cultured as previously described [24]. Briefly, bone marrow cells were obtained from the femoral shafts of 7-week-old male Fisher 344 rats, which were anesthetized and euthanized by exposing of carbon dioxide. The cells were obtained from at least two rats and pooled in order to reduce the influence of individual differences. The culture medium was Eagle’s minimal essential medium (with Earle’s Salt and l-glutamine) containing 15% FBS and antibiotics (100 units/mL penicillin G, 100 µg/mL streptomycin sulfate, and 0.25 µg/mL amphotericin B). The medium was replaced twice a week, and cells at passages 2–3 were used in this study. This study was carried out in strict accordance with the recommendations in the Guide for the Care and Use of Laboratory Animals of the University of Kitakyushu. The protocol was approved by the Committee on the Ethics of Animal Experiments of the University of Kitakyushu.

### Cell culture

A7r5 cells, HeLa cells, and HOS cells were cultured in DMEM supplemented with 10% FBS and antibiotics (100 units/mL penicillin G, 100 µg/mL streptomycin sulfate). The medium was replaced twice a week.

### Cell staining

Cells were seeded in a 35-mm culture dish at around 1 × 10^4^ cells/cm^2^. The cells were fixed with 4% paraformaldehyde and stained with 0.4% trypan blue solution. The cells were imaged at 8.4× magnification using a stereomicroscope (SZX12; Olympus, Tokyo, Japan) equipped with a DP70 color charge-coupled device camera (Olympus).

### Image extraction of cell distribution

The obtained cell images were analyzed using Image J software (NIH, Bethesda, MD). Each cell image was split into each RGB color channel. Then, the red channel image, which was the image with the highest contrast against the background, was subtracted from the background light shadow. The image was binarized with the adequate threshold intensity value, which was determined by referring to the highly magnified image. The size of the square image was dependent on each cell type (rat MSC, 3.5 × 3.5 mm; Hela and HOS cells, 3 × 3 mm; A7r5 cells, 4 × 4 mm). After clipping the square image from the binarized image, the resolution of the image decreased to 100 × 100 px. The clipping cell size was determined by the area of approximately 1 × 10^4^ cells. We prepared more than five images for each condition.

### Cell dynamics simulator

#### Simulator specification

**Fig. 1:**
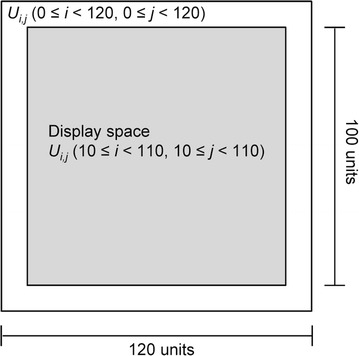
Cell proliferation simulation space. The total calculated space is 120 × 120 cell units. *U*_*i,j*_ (0 ≤ *i* < 120, 0 ≤ *j* < 120) represents each calculation unit. The display space is made up of the central 100 × 100 cell units (*U*_*i,j*_ (10 ≤ *i* < 110, 10 ≤ *j* < 110))

The 2D cell simulator was developed using Java (Oracle, Redwood Shores, CA). The cell distribution of the simulator was modeled by the cellular automata. The calculated space was set to 120 × 120 cell units and the displayed space on the screen was 100 × 100 cell units of the center of the calculated space (Fig. 1). Each unit indicates whether it is occupied by cells (black point) or it is vacant (white point). The model includes cell movement and division regulated by cell–cell interactions (cell–cell adhesion inhibits cell movement and cell–cell contact inhibition inhibits cell division). One cycle of the calculation of cellular events (movement and division) indicates 10 min in a virtual environment. Because cells in the early stages of cell culture exhibit a proliferation lag, we simulated cell proliferation behavior after 24 h of culture. Therefore, the cell seeding number, *s*_*0*_, was used to represent the cell number after 1-day culture in each cell type.

#### Cell dynamics

**Fig. 2:**
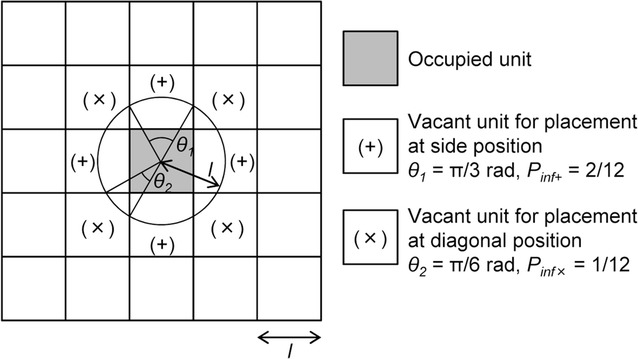
Array of unit squares representing cells. One unit is in contact with the neighboring 8 units. *l* is the length of a unit, which is altered in each cell type. *θ*_*1*_ (π/3) and *θ*_*2*_ (π/6) are the angles which face to the side and the diagonal position of units, respectively. The influence probabilities from the neighboring units are 1/12 at a diagonal position (*P*_*inf*×_) or 2/12 at a side position (*P*_*inf*+_), which is determined by the angle

Each cell unit contacts the neighboring 8 units (Fig. 2). According to a previous study using cellular automata dynamical simulation of cell culture [21], the influence probabilities for the center unit from the neighboring 8 units are 1/12 and 2/12 at a diagonal position (*P*_*inf*×_) and a side position (*P*_*inf*+_) of a center unit, respectively (Fig. 2). Each influence probability was derived from the ratio of the central angle to 2π rad on a circle with a radius equal to the length of one side of the unit square.

**Fig. 3:**
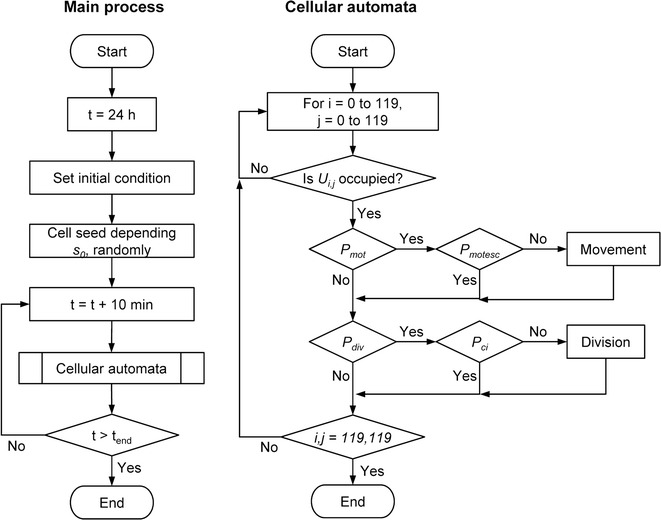
Flow charts of the cell proliferation simulation. The *left chart* shows the main process and the *right chart* shows the cellular automata process. One cycle of the process is 10 min of virtual cell culture. There are two events, namely movement and division, in the cellular automata process. The movement event is started according to the *P*_*mot*_, which is determined by the cell motility parameter *mot*, and terminated according to the *P*_*motesc*_, which is determined by the effect of cell–cell adhesion (parameter *a*) with surrounding cells (the parameter is *P*_*su*_). The cell division event is started according to the *P*_*div*_, which is determined by the cell doubling time *t*_*d*_, and terminated according to the *P*_*ci*_, which is determined by the effect of surrounding cells

Figure 3 shows a flowchart of the cellular automata simulation. The method of cellular automata was applied in all cell occupied units.

The cell movement event is produced according to the probability, *P*_*mot*_, which depends on the cell motility parameter *mot*; *P*_*mot*_ = 1/(6 *mot*). The *mot* means the time (h) for a single unit transfer of the cell on average. During the cell movement event, the cell can escape from the event according to the influence of the surrounding cells. The total influence of surrounding cells, *P*_*su*_, is determined based on the influence probabilities, a diagonal position of cell number *n*_×_, and a side position of cell number *n*_+_, *P*_*su*_ = *n*_×_ *P*_*inf*×_ + *n*_+_ *P*_*inf*+_ = (*n*_×_ + 2*n*_+_)/12.

Using the cell–cell adhesion parameter *a*, the cell escapes from the movement event with the probability, *P*_*motesc*_,

$$P_{motesc} = 1 - \left( {1 - \frac{a}{100}} \right)^{{12P_{su} }} .$$

If the cell does not escape from the event, the cell moves around a vacant unit depending on the influence probabilities.

The cell division event is produced according to the probability, *P*_*div*_, which depends on the cell doubling time (h) *t*_*d*_,

$$P_{div} = \, {\alpha \mathord{\left/ {\vphantom {\alpha {\left( { 6t_{d} } \right)}}} \right. \kern-0pt} {\left( { 6t_{d} } \right)}}_{,}$$

where *α* (=0.7147) is the offset of the cell division due to overlap with the immediately preceding cell division. During the cell division event, the cell can escape from the event according to the influence of the surrounding cells. The total influence is determined by the influence probabilities (*P*_*inf*×_, *P*_*inf*+_) and the cell number of the diagonal position and the side position of the cell, c*i* = *12 n*_×_ *P*_*inf*×_ + *12 n*_+_ *P*_*inf*+_ = *n*_×_ + *2 n*_+_. According to the cell–cell contact inhibition parameters, the cell escapes from the division event with the probabilities *P*_*ci*_. The *P*_*ci*_ can be fixed arbitrarily; but, in this study, we used four different parameters as null (0, 0, 0, 0, 0, 0, 0, 0, 0, 0, 0, 1), weak inhibition (0, 0, 0, 0, 0, 0, 0, 0, 0, 0, 0.9, 1), positive inhibition (0, 0, 0, 0, 0, 0, 0, 0, 0.4, 0.8, 0.95, 1), and strong inhibition (0, 0, 0, 0, 0, 0.3, 0.6, 0.8, 0.9, 0.96, 0.99, 1). If the cell does not escape from the event, one of the daughter cells occupies a surrounded vacant unit depending on the influence probabilities, and the other daughter cell occupies the unit of the original mother cell.

#### Estimation of cell proliferation parameters

The simulated cell proliferation parameters could be estimated from the growth curve and cell proliferation images. Cell proliferation was serially simulated with various cell proliferation parameters. Then, the simulated cell number was evaluated and rated by comparing with the experimentally obtained data using least square analysis. Within the several higher conditions, the most matching parameters were finally determined by visually comparing the simulated cell images with the experimentally obtained cell images.

## Results and discussion

### Analysis of cell proliferation under experimental culture conditions

**Fig. 4:**
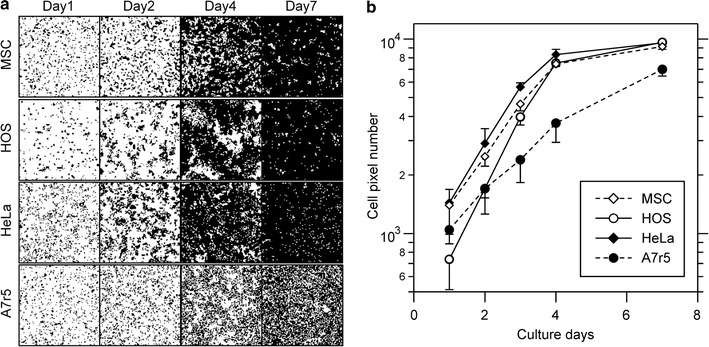
Experimentally obtained cell proliferation images. **a** The cell proliferation images of each cell type. The images were processed as described in the “Methods” section. Each *black point* represents a cell. **b** The growth curves of each cell type. The data shows the average pixel number ± standard deviation, which are calculated from more than five cell images for each condition

First, we analyzed cell proliferation using four different types of cells: rat mesenchymal stem cells (MSCs), human cervical cancer HeLa cells, human osteosarcoma HOS cells, and rat aorta smooth muscle A7r5 cells. These cells were stained with trypan blue daily. The obtained cell images were binarized and clipped into squares of about 10^4^ cells. Then, the resolution of the clipped image was decreased to 100 × 100 px. Figure 4a shows the binarized and clipped images of each cell type. The appearance of the proliferation process varied according to the cell type. The area covered by MSCs showed a mottled pattern at 4 days and cell-free area remained even at 7 days. HOS cells grew in large colonies initially, but eventually showed a confluent pattern. HeLa cells grew rapidly and quickly reached confluence. A7r5 cells showed sparse cell confluence after 7 days of culture. The growth curves, calculated using number of black pixels in the cell images, are shown in Fig. 4b. The cell-stained pixels grew exponentially in the early phase of cell culture. Therefore, the transition of the pixel number represents the cell proliferation curve. Next, we calculated the doubling time and the pixel number at 24 h for each cell type using the pixel proliferation data from the early phase of the cell culture. The calculated doubling times of MSCs, HOS, HeLa, and A7r5 cells were approximately 27.7, 19.7, 24.2, and 40.3 h, respectively. The pixel number at 24 h for each cell type was 1390, 740, 1450, and 1070, respectively. Using these experimental data, we analyzed the cell–cell interaction properties of each cell type.

### Development of cell proliferation simulator

**Fig. 5:**
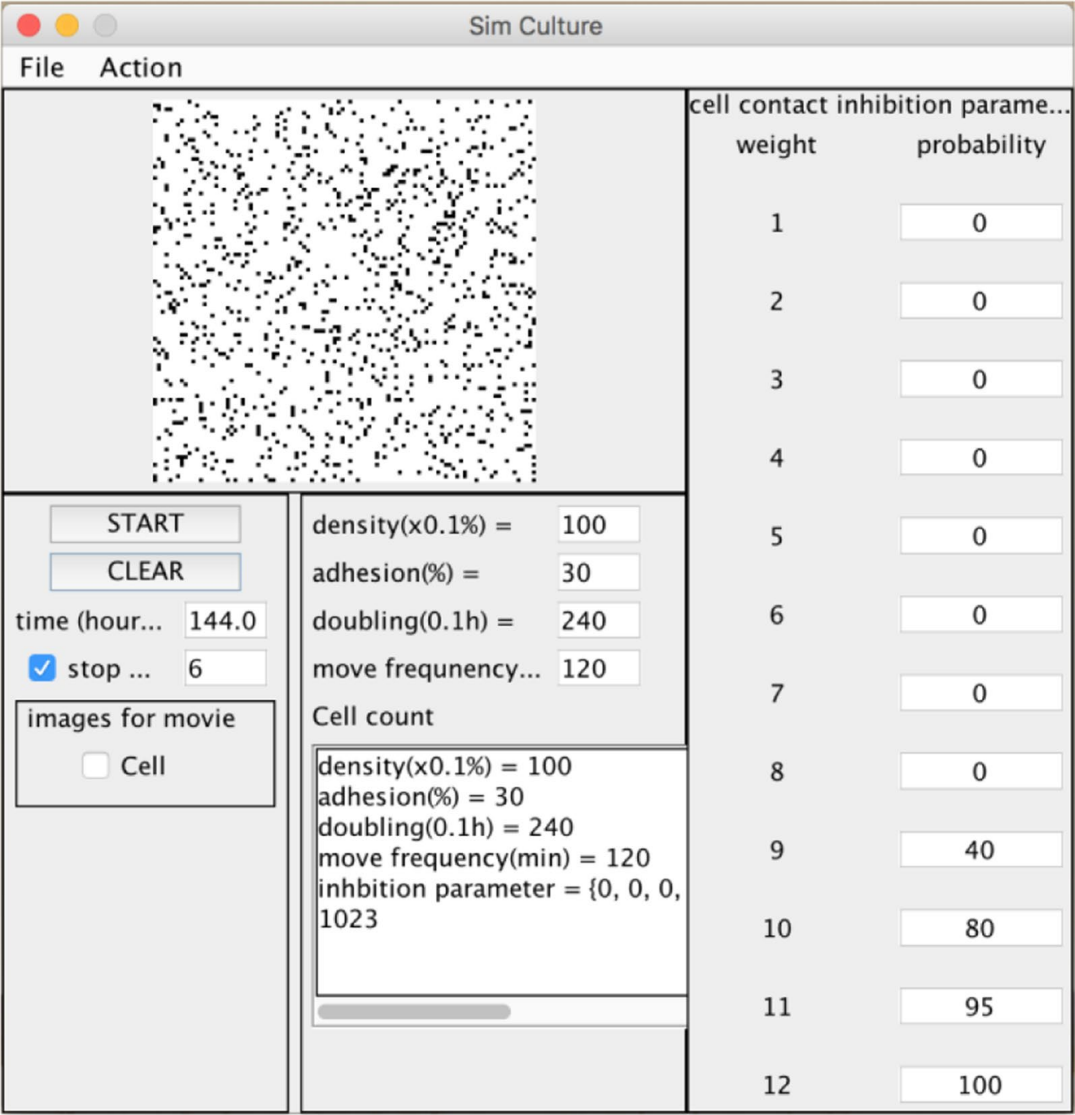
Graphical user interface (GUI) of the simulator. Proliferating cell image and cell numbers are uploaded on the screen. All parameters can be uploaded to the GUI directly

To estimate cell–cell interaction properties such as cell–cell adhesion and cell–cell contact inhibition, we constructed a simple cell proliferation simulator using a cellular automata model. Here, cell–cell adhesion and cell–cell contact inhibition show cell migration inhibition and cell division inhibition, respectively. The graphical user interface of our developed system is shown in Fig. 5. We arranged 120 × 120 cell units of virtual space as cell proliferation space (Fig. 1), and a single black point was used to represent a single cell (Fig. 2). We assumed that the centered 100 × 100 cell units of the virtual space reflected the binarized and clipped images of cultured cells (Fig. 4a). To construct the simulator, we set 5 parameters, which were initial cell seeding cell number *s*_*0*_, doubling time *t*_*d*_ (h), motility *mot* (h), cell–cell adhesion *a* (%), and cell–cell contact inhibition *P*_*ci*_ (detailed in “Methods”).

**Fig. 6:**
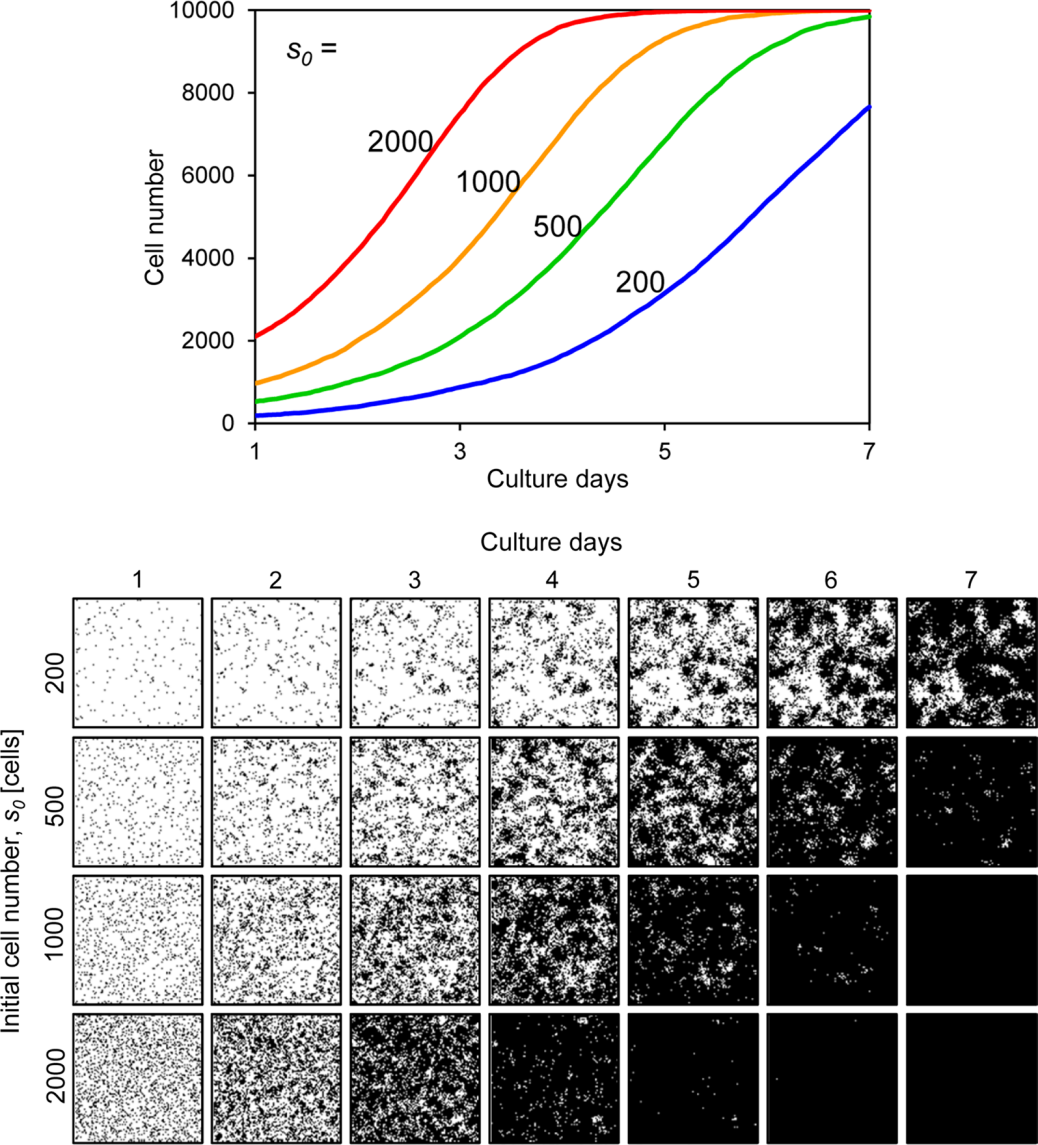
Cell proliferation simulation with varying initial cell numbers. The *upper graph* shows the growth curves and the *lower images* are the proliferating cell images for each condition. The initial cell number *s*_*0*_ ranged from 200 to 2000. Other parameters were constant values: *t*_*d*_, 24; *a*, 20; *mot*, 4; *P*_*ci*_, weak

**Fig. 7:**
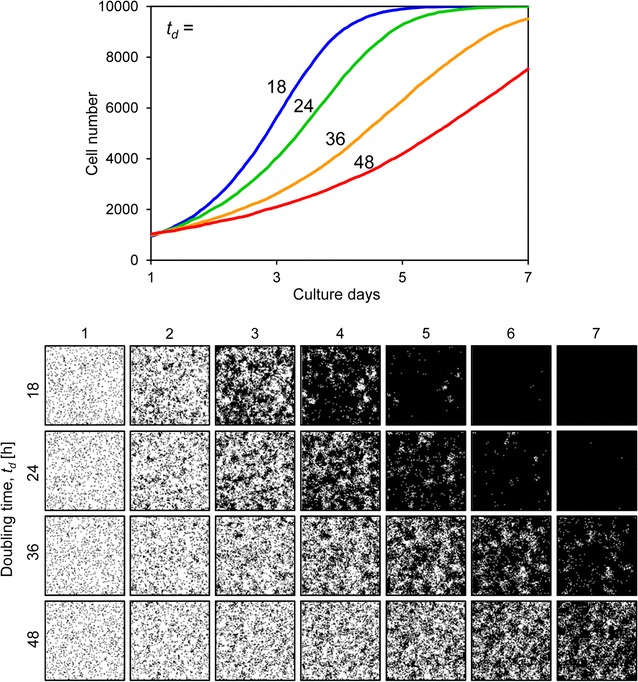
Cell proliferation simulation with varying cell doubling time. The *upper graph* shows the growth curves and the *lower images* are proliferating cell images for each condition. The doubling time *t*_*d*_ [h] ranged from 18 to 48. Other parameters were constant values: *s*_*0*_, 1000; *a*, 20; *mot*, 4; *P*_*ci*_, weak

**Fig. 8:**
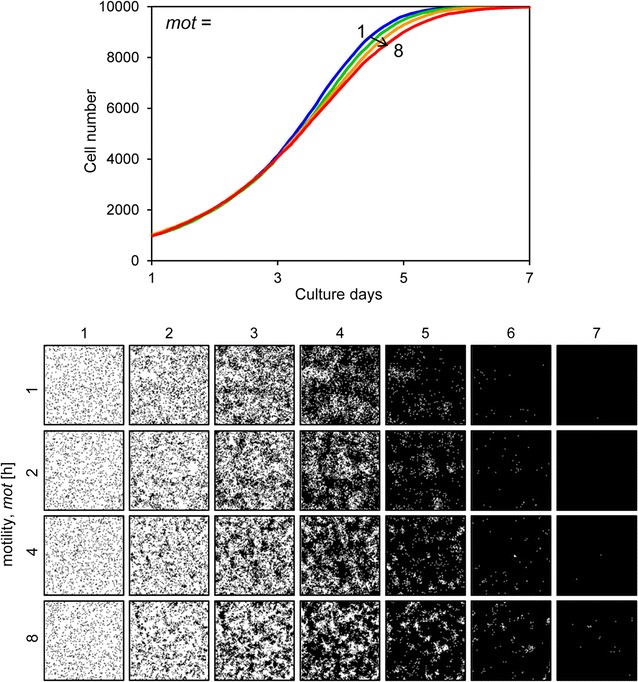
Cell proliferation simulation with varied cell motility. The *upper graph* shows the growth curves and the *lower images* are proliferating cell images for each condition. The motility *mot* (h) ranged from 1 to 8. Other parameters were constant values: *s*_*0*_, 1000; *t*_*d*_, 24; *a*, 20; *P*_*ci*_, weak

**Fig. 9:**
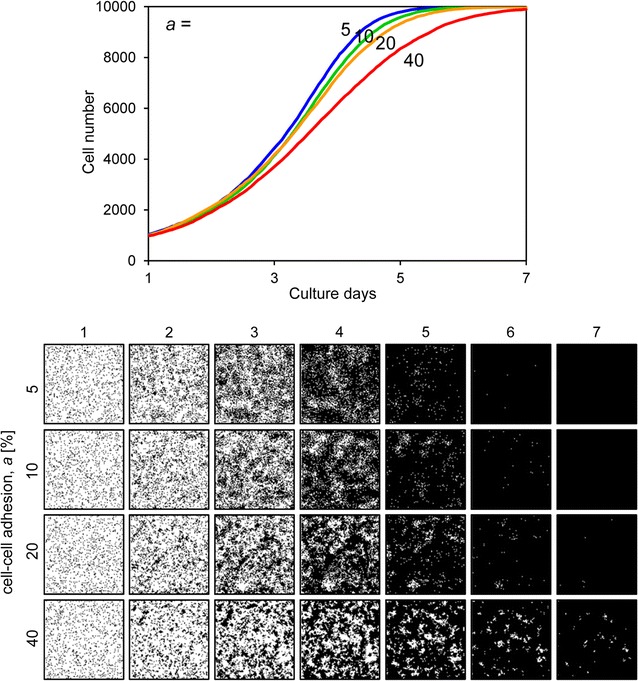
Cell proliferation simulation with varied cell–cell adhesion. The *upper graph* shows the growth curves and the *lower images* are proliferating cell images for each condition. The cell–cell adhesion *a* (%) ranged from 5 to 40. Other parameters were constant values: *s*_*0*_, 1000; *t*_*d*_, 24; *mot*, 4; *P*_*ci*_, weak

**Fig. 10:**
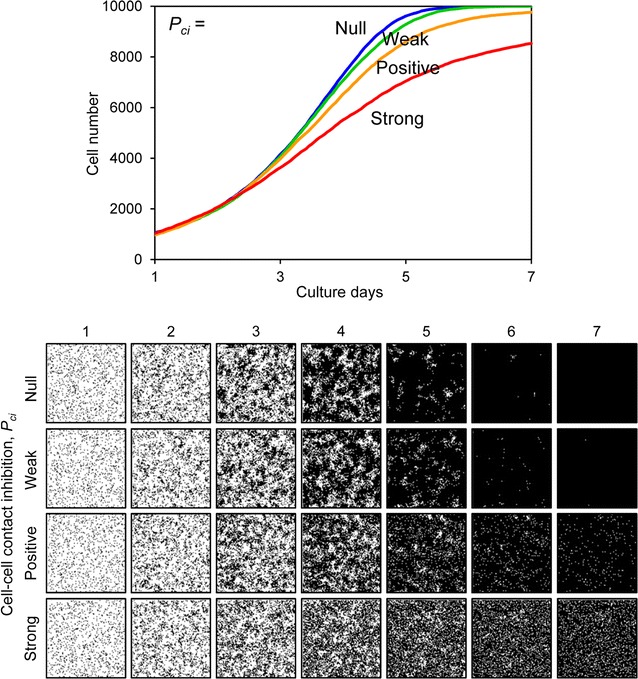
Cell proliferation simulation with varied cell–cell contact inhibition of proliferation. The *upper graph* shows the growth curves and the *lower images* are proliferating cell images for each condition. The cell–cell contact inhibition *P*_*ci*_ ranged from null to strong. Other parameters were constant values: *s*_*0*_, 1000; *t*_*d*_, 24; *mot*, 4; *a*, 20

First, we showed outputs from our simulator. The outputs of this simulator were the cell number and cell image for each culture time, based on setting the above five parameters. Figure 6 shows results when the initial cell number *s*_*0*_ was variable. The growing cells gradually filled the cell culture space, eventually occupying the entire space (Fig. 6). Comparing low versus high *s*_*0*_ values, the cells with low *s*_*0*_ values grew and formed a large cell colony, whereas cells with high *s*_*0*_ values completely filled the space (Fig. 6). Figure 7 shows results when the doubling time *t*_*d*_ was variable. When *t*_*d*_ was set to 18 h, the cells occupied almost the entire culture space on day 5 of culture. However, when *t*_*d*_ was set to 48 h, the slope of the growth curve decreased and the cells could not fill the space by day 7 (Fig. 7). Figure 8 shows the results when the motility *mot* was variable. The growth curves demonstrate gradual growth when the *mot* value was high, i.e. low motility conditions (Fig. 8). Focusing on the growing cell images at day 4 of culture, the cells were well spread when the *mot* value was low, whereas they displayed a mottled pattern when the *mot* value was high (Fig. 8). Figure 9 shows the results when cell–cell adhesion *a* was variable. The slope of the growth curve gradually decreased when the value of *a* was high (Fig. 9). The cell images obtained after 4 days of culture showed a highly mottled pattern when the value of *a* was high (Fig. 9). Figure 10 shows the results when cell–cell contact inhibition *P*_*ci*_ was variable. When the *P*_*ci*_ was set as null, the cells completely filled the space after 7 days of culture (Fig. 10). However, when the *P*_*ci*_ was set to strongly inhibited conditions, the cells proliferated but only sparsely filled the culture space (Fig. 10).

The initial cell number *s*_*0*_ and the doubling time *t*_*d*_ are critical factors for cell proliferation, and when they are changed, the form and slope of the growth curve drastically changes (Figs. 6, 7). In this study, these two parameters were determined by the experimental results. However, the effects of motility *mot*, cell–cell adhesion *a*, and cell–cell contact inhibition *P*_*ci*_ were relatively restricted. These parameters affected growing cell patterns (Figs. 8, 9, 10). In this study, we could not determine these parameters from the experimentally obtained results. Of these parameters, *mot* and *a* are parameters for assessing for the cell movement event (Fig. 3). Comparing the cell proliferation simulation with variable *mot* to that with variable *a*, the impact of *a* was more significant than that of *mot* (Figs. 8, 9). Furthermore, cell motility can be determined by some other methods [7, 25]. Therefore, in this study, we simulated the cell proliferation behavior with the cell–cell adhesion *a* and cell–cell contact inhibition *P*_*ci*_, which are the parameters reflecting the cell–cell interaction activity, as variable.

### Simulation of experimentally obtained cell proliferation

One of the advantages of simulation is obtaining hypothetical experimental data. We can serially simulate various cell proliferation behaviors with varied cell proliferation parameters. Therefore, we attempted to estimate the cell–cell interaction activity parameters, i.e. cell–cell adhesion *a* and cell–cell contact inhibition *P*_*ci*_, for MSCs, HOS, HeLa, and A7r5 cells. The experimentally obtained initial cell number and doubling time of each cell were used in the simulation. The motility was defined as a constant value. *a* varied from 10 to 40, in intervals of 10. *P*_*ci*_ was defined as null, weak, positive, or strong inhibition. The simulated data were evaluated by comparison with experimentally obtained data. The simulated growth curves were ranked by least square analysis with the experimental growth curves. After choosing several high-ranking conditions, we visually compared the simulated cell images with experimentally obtained images, and then determined the simulation parameters of each cell.

**Fig. 11:**
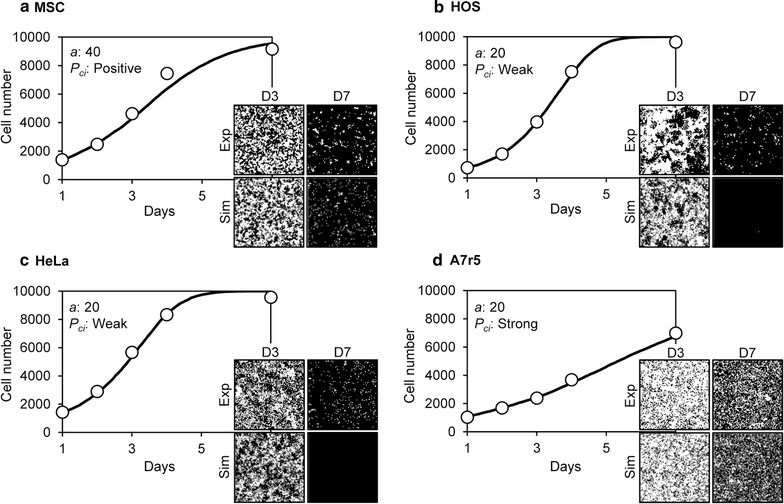
Cell proliferation simulation for each cell type. The cell types are MSC (**a**), HOS (**b**), HeLa (**c**), and A7r5 (**d**). The simulated and experimentally obtained cell proliferation results are shown. The simulation growth curves are represented by *lines* and those from experimentally obtained results are shown as *circles*. The simulated cell images (Sim) and experimentally obtained images (Exp) are shown for day 3 (D3) and 7 (D7). The initial cell number *s*_*0*_ and doubling time *t*_*d*_ were used as determined in Fig. 4. The motility *mot* was used at a constant value of 4 (h). Cell–cell adhesion *a* and cell–cell contact inhibition *P*_*ci*_ values used are indicated for each graph

Figure 11 shows the simulated cell proliferation behaviors of each cell type. The simulated growth curves fit with the experimental data for each cell type (Fig. 11). With regard to the cell growth images, simulated images roughly resembled the experimental images for each cell type. Simulated MSCs demonstrated a high cell–cell adhesion (*a* = 40) and positive cell–cell contact inhibition (Fig. 11a). These cells exhibited fine mottled patterns of cell growth at day 3, and they did not reach confluence at day 7 (Fig. 11a). The fine mottled pattern was similar to that observed in the experimentally obtained cell proliferation images. Simulated HOS cells showed low cell–cell adhesion (*a* = 20) and weak cell–cell contact inhibition (Fig. 11b). The simulated growth curve highly correlated with the experimental results. However, the experimental HOS cells exhibited a large colony cell pattern at day 3, but the simulation could not reproduce the cell patterns (Fig. 11b). The simulated HeLa cells demonstrated low cell–cell adhesion (*a* = 20) and weak cell–cell contact inhibition (Fig. 11c). The simulated growth curve and cell images correlated with the experimental results (Fig. 11c). The simulated A7r5 cells demonstrated low cell–cell adhesion (*a* = 20) and strong cell–cell contact inhibition (Fig. 11d). The simulated growth curve of A7r5 cells also correlated with experimentally obtained results. The simulated cell images showed sparse cell confluence with many vacant cell spaces at the later stages of cell culture, which was consistent with the experimentally obtained A7r5 cell pattern at day 7 (Fig. 11d).

**Fig. 12:**
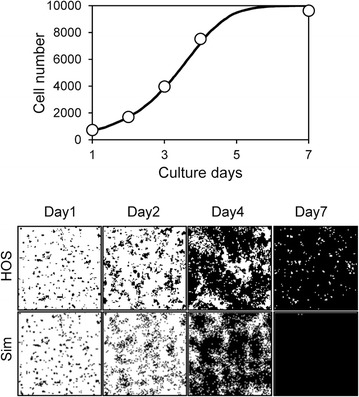
Cell proliferation simulation of HOS cells using the experimentally obtained initial cell pattern. The *upper graph* shows the growth curves and the *lower images* are simulated (*line* and Sim) and experimentally obtained (*circles* and HOS) proliferating cell images. The doubling time *t*_*d*_ was used as determined in Fig. 4. Other parameters were: *mot*, 4; *a*, 10; *P*_*ci*_, weak

In our system, the initial cell number and cell doubling time were experimentally obtained for each cell type. These two parameters most significantly affected the cell growth curve, and thus our simulated growth curves highly correlated with those that were experimentally obtained (Fig. 11). However, simulated images of growing cells were not always consistent with the experimentally obtained images. Cell–cell adhesion affected the fine mottled formation of cell patterns, and cell–cell contact inhibition affected cell confluency at a later stage of cell culture (Figs. 9, 10). Therefore, we successfully simulated MSCs, which formed fine mottled patterns; HeLa cells, which demonstrated global proliferation; and A7r5 cells, which reached highly sparse confluence (Fig. 11). However, we could not fully simulate HOS cell proliferation, which demonstrated a large colony growth pattern (Fig. 11). The formation of large colonies requires a small number of initial seeding cells (Fig. 6). However, the initial cell number of HOS cells was about 740 (Table 1), which is higher than the initial cell number forming the large cell colony (Fig. 6). This inconsistency is attributable to the cell attachment behavior to the culture substrate. The experimentally obtained cell images of HOS cells at day 1 showed many small cell aggregations that contain several cells (Fig. 4). The cell-seeding algorithm of our system was assigned a random number. Thus, the simulated initial cell location was more dispersed than the experimental HOS cell initial location. We then simulated the proliferation of HOS cells using the experimentally obtained cell image from day 1, and repeated the cell simulation with cell–cell adhesion *a* as variable and weak cell–cell contact inhibition. With low cell–cell adhesion (*a* = 10), the simulated growth curve correlated with the experimental growth curve of HOS cells (Fig. 12). Furthermore, the simulated cell image showed large cell colony forming proliferation (Fig. 12). Therefore, the cells that attached to the substrate with small cell aggregation were well simulated using the experimentally obtained initial cell image.

**Table 1:** Parameters of each cell proliferation simulation

Parameter	MSC	HOS	HeLa	A7r5
*s*_*0*_ (cells)	1390	740	1450	1070
*t*_*d*_ (h)	27.7	19.7	24.2	40.3
*a* (%)	40	10	20	20
*P*_*ci*_	Positive	Weak	Weak	Strong

A summary of the simulated parameters for each cell type is shown in Table 1. From our simulation, we identified three modes of cell proliferation behavior based on the four cell types analyzed. The first mode includes cancerous HOS and HeLa cells, which exhibit low cell–cell adhesion and weak cell–cell contact inhibition. The second was observed in MSCs, which exhibit high cell–cell adhesion and positive cell–cell contact inhibition. The third was observed in A7r5 cells, which exhibit low cell–cell adhesion and strong cell–cell contact inhibition. The cancer cells demonstrated exponential proliferation and reached more than 95% confluence (Fig. 4). Generally, cancer cells can grow without any positive cell–cell adhesion or cell–cell contact inhibition, and they can easily attain an over-confluent cell pattern. Therefore, our simulated results reasonably describe these cells. The MSCs demonstrated a fine mottled proliferation pattern (Fig. 4), which requires high cell–cell adhesion values to be produced by the simulation (Fig. 9). Mesenchymal cells exhibit cell–cell contact inhibition and intercellular adhesive force is reported to be high [16]. Therefore, we concluded that the selected value of the cell–cell adhesion of MSCs was reasonable. A7r5 cells showed a very sparse confluence pattern (Fig. 4). These sparse patterns require strong cell–cell contact inhibition. On the other hand, simulated A7r5 cells did not demonstrate high cell–cell adhesion. This is likely because the growth curve of A7r5 cells was exponential and the proliferative cell pattern did not show a mottled pattern. Thus, we suppose that this is the interaction properties of A7r5 cells.

In this study, we simulated all cells with a constant motility (*mot* = 4.0). This is because the effect of motility was limited in the simulated growth curve (Fig. 8). It is difficult to determine appropriate parameter values when the number of variables increases. Cell simulated results, in particular, can be quantitatively compared to experimentally obtained cell growth curves, but only qualitatively compared to experimentally obtained cell proliferation patterns. Therefore, it is necessary to reduce the number of variables. In this study, we treated only two parameters, cell–cell adhesion and cell–cell contact inhibition, as variables, and we succeeded with cell proliferation simulations (Figs. 11, 12). Future studies on the quantitative comparison of simulated cell images and experimentally obtained images are needed. However, it is difficult to evaluate the similarity of two different images. Here, we attempted 2D Fourier transformation of cell images and compared the power spectrums of the resulting images, but we did not obtain quantitative values from the comparison. Therefore, another image comparison algorithm will be needed for this type of analysis.

## Conclusions

We developed a cellular automata simulator for determining the cell–cell interaction properties of cultured cells. Cultured cells proliferated with their characteristic cell patterns and growth curves. We determined their doubling time from their growth curves, and then simulated their proliferation using their doubling times and initial cell numbers, with cell–cell interaction properties as variables. The proliferation could be simulated and we could determine their cell–cell interaction properties. HOS and HeLa cancer cells exhibited low cell–cell adhesion and weak cell–cell contact inhibition of proliferation. MSCs exhibited high cell–cell adhesion and positive cell–cell contact inhibition of proliferation. A7r5 cells exhibited low cell–cell adhesion and strong cell–cell contact inhibition of proliferation. These cell–cell interaction properties, which are characteristic features of each cell, could not be determined from typical cell culture experiments. Therefore, our developed simulation approach is an easy method for evaluating the cell–cell interaction properties of cells. Furthermore, this simulation can be used as a basic system for simulating many types of cellular events.

## Abbreviations

HOS: human osteosarcoma
MSC: mesenchymal stem cell
2D: 2 dimensional
FBS: fetal bovine serum
GUI: graphical user interface
